# The Impact of Prescription Barriers to Special Low Protein Foods and Protein Substitutes on PKU Care: Findings from a National Survey of Metabolic Dietitians

**DOI:** 10.3390/nu18183092

**Published:** 2026-09-21

**Authors:** Martina Tosi, Suzanne Ford, Anne Grimsley, Sarah Bailey, Anita MacDonald

**Affiliations:** 1 Department of Dietetics, Birmingham Women’s and Children’s Hospital, Birmingham B4 6NH, UK; martina.tosi@nhs.net; 2 National Society for Phenylketonuria (NSPKU), Sheffield S12 9ET, UK; suzanne.ford@nspku.org; 3 Department of Nutrition & Dietetics, Bristol NHS Foundation Trust, Bristol Royal Infirmary, Bristol BS2 8HW, UK; 4 Royal Belfast Hospital for Sick Children, 274 Grosvenor Rd, Belfast BT12 6BA, UK; anne.grimsley@belfasttrust.hscni.net; 5 All Wales Inherited Metabolic Disease Service, University Hospital of Wales, Heath Park Way, Cardiff CF14 4XW, UK; sarah.bailey3@wales.nhs.uk

**Keywords:** phenylketonuria, special low protein foods, protein substitutes, prescription, general practitioner, home delivery, pharmacy, dietitian

## Abstract

**Background/Objectives**: Timely access to protein substitutes and special low-protein foods (SLPFs) is essential for effective dietary management of phenylketonuria (PKU). As key healthcare professionals responsible for coordinating and delivering nutritional therapy, metabolic dietitians are directly involved in managing prescription-related issues that may arise within the access pathway. This study aimed to identify the main prescription-related barriers experienced by metabolic dietitians in the UK and to explore their implications for patient care, clinical workload, and the delivery of specialist PKU services. **Methods**: A structured questionnaire was distributed by the National Society for Phenylketonuria to dietitians involved in the care for individuals with PKU in the UK. Anonymised responses were analysed descriptively to identify the types, frequency, and impact of prescription-related issues. **Results**: A total of 448 responses were received from metabolic dietitians across the UK reporting prescription-related problems affecting dietary products. Most reported incidents involved children (306/446; 68.6%). Among responses specifying the affected product category (*n* = 443), SLPFs accounted for most reported problems (261/443; 58.9%), followed by protein substitutes (120/443; 27.1%) and finally combined protein substitute and SLPF issues (62/443; 14.0%). Qualitative analysis identified barriers across all stages of the prescribing pathway, with issues primarily relating to GP prescribing processes, home delivery and supply, pharmacy services, and broader administrative and system-related factors. These disruptions affected continuity of dietary management and metabolic care, with reported consequences including reduced access to prescribed dietary products, difficulties maintaining dietary adherence, increased patient and family burden, anxiety and frustration, and a substantial hidden workload for metabolic dietetic services. **Conclusions**: Prescription-related problems represented a significant barrier to timely access to essential medical foods for individuals with PKU in the UK. The findings highlight challenges across the prescribing and supply pathway that compromise access to both protein substitutes and SLPFs, impose additional burdens on patients and families, and increase workload for specialist metabolic dietetic services. Addressing these system-level barriers, alongside strengthening communication, coordination, and prescribing processes across stakeholders, may improve continuity of dietary treatment and enable more efficient delivery of specialist metabolic care.

## 1. Introduction

Phenylketonuria (PKU; OMIM: 261600) is an autosomal recessive disorder of phenylalanine (Phe) metabolism resulting from deficient phenylalanine hydroxylase activity. Untreated hyperphenylalaninaemia causes progressive neurotoxicity leading to intellectual disability, neurological impairment, and psychiatric morbidity. Universal newborn screening and early dietary treatment have transformed outcomes, enabling most individuals to achieve normal cognitive development provided metabolic control is maintained throughout life [1]. Consequently, the principal management is lifelong dietary management with the primary objective of maintaining blood Phe concentrations within recommended therapeutic targets [1,2,3].

Dietary treatment is complex and relies on three interdependent components: restriction of natural protein intake to limit Phe exposure, supplementation with low-Phe protein substitutes to provide safe protein equivalent and essential micronutrients, and the use of specialised low-protein foods (SLPFs) to increase dietary variety, provide energy needs, and support long-term adherence [1,4,5]. These specialised medical foods are not optional adjuncts but essential therapeutic interventions requiring continual adjustment throughout life in response to changes in age, growth, nutritional requirements, metabolic control, lifestyle, and personal food preferences [2].

Metabolic dietitians are central to the delivery of dietary treatment, undertaking specialist nutritional assessment, translating biochemical results into individualised dietary prescriptions, delivering patient and caregiver education, and undertaking ongoing monitoring and adjustment of treatment within multidisciplinary metabolic services [6]. Effective nutritional management therefore depends not only on clinical expertise but also on uninterrupted access to protein substitutes and SLPFs, enabling dietitians to implement and modify individualised treatment plans according to changing clinical needs.

Access to suitable medical foods in the United Kingdom (UK) is dependent on coordination between specialist metabolic services, general practitioners (GPs), primary care prescribing teams, community pharmacies, and home-delivery providers [7,8]. The resulting multistep pathway can complicate continuity of supply, with prescribing errors, poor communication, administrative delays, and supply interruptions reported across the system [8,9,10,11]. These difficulties are amplified by the need to regularly update prescriptions. For individuals receiving several products from different dispensing providers, coordinating prescriptions and deliveries introduces an additional layer of complexity.

Disruptions within this pathway have implications beyond delays in product supply. Inconsistent access to suitable medical foods can undermine individualised dietary management, compromise adherence and metabolic stability, and divert metabolic dietetic time from direct patient care [7]. Resolving prescribing and supply problems frequently requires coordination with GP practices, prescribing teams, pharmacies, suppliers, and families, generating a substantial but largely unrecognised workload for dietitians. This administrative burden reduces capacity for core specialist activities, including nutritional assessment, dietary education, metabolic monitoring, and service development [12].

Despite the pivotal role of metabolic dietitians in coordinating lifelong nutritional management, little is known about their experiences of navigating the UK prescribing and supply pathway for protein substitutes and SLPFs. Existing literature has largely focused on patient and caregiver experiences of accessing prescribed nutritional products, with limited attention given to the healthcare professionals responsible for coordinating prescribing and ensuring continuity of dietary treatment [7,13,14,15]. This represents an important gap, as prescribing and supply problems may arise across multiple interfaces within the healthcare system and require substantial coordination by specialist dietetic teams. Importantly, the barriers encountered by metabolic dietitians may extend beyond individual prescribing difficulties and reflect wider system-level challenges involving primary care, community pharmacies, suppliers, and administrative processes. Understanding these barriers is essential to identify inefficiencies within the current prescribing pathway, determine their consequences for patient care, continuity of treatment, and dietetic workload, and inform opportunities for service redesign to improve access to essential medical nutrition. To our knowledge, no previous UK study has systematically examined access to both protein substitutes and SLPFs for people with PKU from the perspective of metabolic dietitians directly involved in prescribing and coordinating their supply.

The aim of this study was therefore to examine the experiences of UK metabolic dietitians involved in prescribing protein substitutes and SLPFs for people with PKU; identify the principal barriers encountered in routine clinical practice; assess their impact on patient management, continuity of treatment, and dietetic workload; and identify opportunities to improve the efficiency and reliability of the prescribing and supply pathway.

## 2. Materials and Methods

### 2.1. Questionnaire Development

We conducted a national cross-sectional survey of UK metabolic dietitians involved in the dietary management of PKU. Metabolic specialist dietitians were selected as the target respondents because of their central role in the nutritional management of individuals with PKU and their direct involvement in coordinating access to prescribed specialist dietary products. Their role places them at the interface between specialist metabolic services, primary care, community pharmacies, suppliers, and home-delivery providers, providing first-hand experience of prescribing and supply difficulties and their resolution across different stages of the pathway. A structured questionnaire was developed to characterise prescribing and supply issues encountered during routine clinical practice. The questionnaire comprised five items, including two closed-ended questions and three open-ended questions, capturing the type of prescribing issue, the dietary products involved, the stage of the prescribing pathway affected, and the perceived impact on individuals with PKU, caregivers, and metabolic dietetic services. The complete questionnaire is provided in Table A1.

### 2.2. Questionnaire Distribution

The questionnaire was distributed by the National Society for Phenylketonuria (NSPKU) to metabolic dietitians providing specialist PKU care across the UK. Participants were invited to report prescribing and supply problems encountered in routine clinical practice and describe their perceived consequences for patients, families, and metabolic services. Each questionnaire response related to a single prescribing or supply problem encountered in clinical practice. Dietitians were permitted to complete the questionnaire on multiple occasions, with each submission documenting a separate problem. Consequently, the number of questionnaire responses represents the number of individual prescribing or supply problems reported rather than the number of participating dietitians. Data were collected between December 2025 and June 2026.

### 2.3. Data Analysis

Quantitative data were summarised using descriptive statistics. Categorical variables were summarised as frequencies and percentages, with the denominator reported for each analysis where applicable. As each questionnaire response referred to a single prescribing or supply problem, the number of reported incidents represents the number of individual problems reported and does not correspond to the number of individual respondents. No inferential statistical analyses were performed, as the study was descriptive in nature and was not designed to assess associations or differences between groups. Responses to open-ended questions were analysed using conventional content analysis to identify recurring barriers and themes related to prescribing and supply pathways. Data relating to protein substitutes and SLPFs were analysed separately. Where responses referred to both product types, relevant text was assigned to each category according to the issue described. Responses were inductively coded, with related codes grouped into overarching themes and subthemes. The thematic structure was reviewed iteratively for consistency and conceptual overlap, and representative quotations were selected to illustrate the principal barriers identified.

### 2.4. Ethics

This study analysed anonymised responses from healthcare professionals collected as part of a service evaluation of the UK PKU prescribing pathway. Participation was voluntary, and completion and submission of the questionnaire were considered to indicate consent to participate. No patient-level data or personally identifiable information were collected. In accordance with UK Health Research Authority guidance, formal Research Ethics Committee approval was not required because the evaluation involved healthcare professionals reporting their experiences of service delivery and did not involve the collection or analysis of patient-level data.

## 3. Results

A total of 448 prescribing-related incidents were reported by UK metabolic dietitians. Most incidents involved children (306/446, 68.6%), with fewer affecting adults (137/446, 30.7%) or both age groups (5/446, 0.7%). Among the 443 reports specifying product type, SLPFs accounted for most incidents (261/443, 58.9%), followed by protein substitutes (120/443, 27.1%) and issues affecting both product categories (62/443, 14.0%). Problems involving both protein substitutes and SLPFs were reported across paediatric and adult services, with the majority arising in paediatric services (33/62, 54.1%).

Among the 443 reports included in the analysis of the prescription pathway (*n* = 443), the most frequently identified barriers across the prescription pathway were related to GP prescribing (139/443, 31.4%) and home delivery and supply processes (134/443, 30.2%). Pharmacy-related barriers and administrative and system-level barriers each accounted for 85/443 (19.2%) of reported issues.

Among protein substitute incidents (120/443, 27.1%), GP prescribing issues were the most common primary barrier (41/120, 34.2%), followed closely by home delivery and supply issues (40/120, 33.3%). Pharmacy-related barriers accounted for 22/120 (18.3%) of incidents, while administrative and system-level barriers represented 17/120 (14.2%).

For reported issues relating to SLPFs (261/443, 58.9%), home delivery and supply processes were the most frequently identified barrier (84/261, 32.2%), followed by GP prescribing issues (82/261, 31.4%). Pharmacy-related barriers accounted for 56/261 (21.5%) of reported issues, and administrative and system-level barriers represented 39/261 (14.9%).

Among incidents affecting both protein substitutes and SLPFs (62/443, 14.0%), administrative and system-level barriers were the most frequently identified category (29/62, 46.8%). GP prescribing and prescription management issues accounted for 16/62 (25.8%) of incidents, while home delivery and supply-related barriers and pharmacy-related barriers represented 10/62 (16.1%) and 7/62 (11.3%), respectively.

### 3.1. Issues Related to Protein Substitutes

Dietitians identified recurrent failures throughout the protein substitutes prescribing pathway, across primary care prescribing, community pharmacy dispensing, home-delivery services, and communication between organisations. Common problems included delayed or missing prescriptions, incorrect products or quantities, prescribing errors, inappropriate routing of prescriptions, and reluctance by some GP practices to prescribe specialist nutritional products. Pharmacy-related barriers included difficulties obtaining products despite manufacturer availability, stock shortages, dispensing errors, and limited familiarity with specialist metabolic products. Home-delivery failures, including delayed, incomplete or incorrect deliveries and inflexible ordering systems, further disrupted continuity of supply.

### 3.2. Issues Related to Special Low-Protein Foods

Barriers affecting SLPFs were similar to protein substitutes but occurred more frequently and reflected the greater complexity of supplying a wide range of specialist food products. Respondents reported delayed or missing prescriptions, dispensing errors, communication failures, difficulties accessing products through community pharmacies, and repeated disruptions to home-delivery services. Product quality issues, including damaged or unusable foods following delayed delivery, and delivery of short shelf-life products were frequently described. There were challenges associated with ordering systems that included limited flexibility, unclear ordering processes and difficulties adapting orders when dietary requirements changed. Respondents reported that patients and responders were commonly unclear about supply routes of SLPFs.

Primary care prescribing represented a major source of disruption. Respondents described delayed or omitted prescriptions, incorrect quantities, missing products, and failure to implement recommendations from specialist metabolic teams. Frequently affected products included staple foods such as low-protein bread, pasta, and milk replacements. Some respondents also reported reluctance among GP practices to prescribe SLPFs because of perceived costs or limited understanding of their therapeutic role in PKU management.

Dispensing errors involving incorrect products or quantities, together with delayed, incomplete or inaccurate home deliveries, further disrupted continuity of supply.

### 3.3. Clinical Consequences of Supply Disruptions to Protein Substitute and Specialist Low Protein Foods

Disruptions in access to protein substitutes compromised implementation of prescribed dietary treatment and were reported to adversely affect both metabolic control and nutritional management. Respondents described interrupted or reduced protein substitute intake, difficulties achieving prescribed protein requirements, and delays in implementing clinically recommended dietary changes. These interruptions were associated with deterioration in metabolic control, including increased blood Phe concentrations, while some respondents reported delayed blood sampling because patients anticipated unfavourable metabolic results.

Reduced access to protein substitutes also disrupted established dietary routines, limiting dietary flexibility, reducing nutritional adequacy, and increasing reliance on less suitable alternatives. Once protein substitute intake had been interrupted, respondents reported that re-establishing adherence was often challenging, resulting in prolonged disruption to dietary management. Particular concern was expressed for infants and young children, individuals with classical PKU, and women planning pregnancy or during pregnancy.

Interruptions in access to SLPFs similarly compromised adherence to prescribed low-Phe diets by reducing dietary variety, limiting meal planning, and increasing hunger, particularly among children dependent on staple low-protein foods. Restricted access to SLPFs also reduced dietary flexibility and made it more difficult to sustain long-term adherence to dietary treatment.

Across both categories of medical foods, recurrent interruptions in access contributed to a sense of treatment insecurity and undermined confidence in the ability to meet prescribed dietary goals. Respondents perceived unreliable access to essential prescribed dietary products as a barrier to effective clinical management, potentially eroding motivation for lifelong dietary adherence and increasing the risk of suboptimal metabolic control.

**Figure 1:**
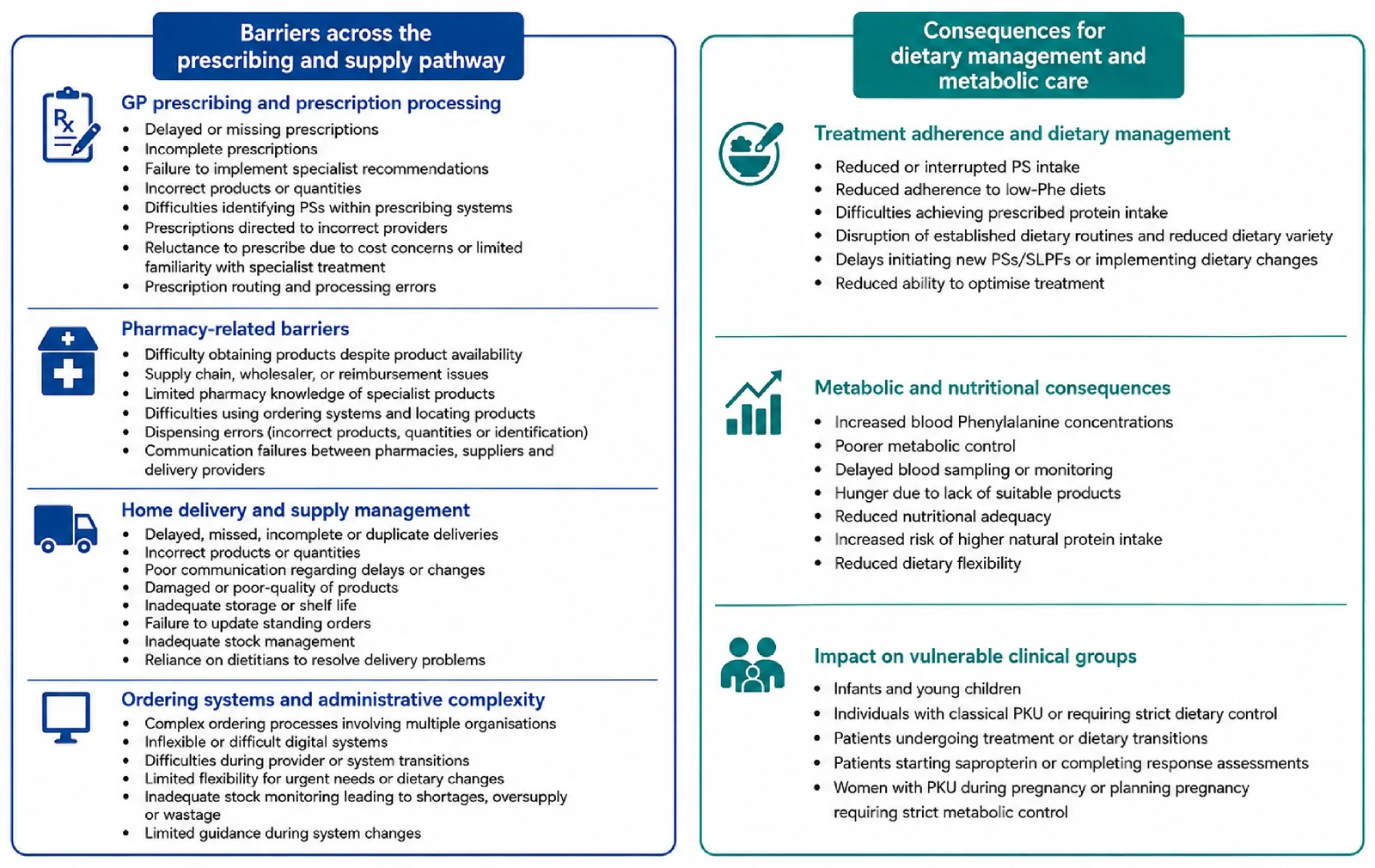
Barriers to accessing medical foods in PKU and consequences for dietary management and metabolic care (figure created by the authors using BioRender and refined using AI-assisted visual editing).

Figure 1 provides an overview of the main themes identified regarding barriers to accessing medical foods and consequences for dietary management and metabolic care.

### 3.4. Impact on Patients and Families of Supply Disruptions to Protein Substitute and Specialist Low Protein Foods

Recurrent disruptions in access to medical foods had substantial psychological, practical, and social consequences for individuals with PKU and their families. Respondents consistently described persistent anxiety arising from uncertainty over the availability of essential dietary treatments, particularly before weekends, public holidays, travel, and pregnancy, when uninterrupted supply was essential. Families caring for young children or individuals with autism, learning disabilities, complex social circumstances or other additional support needs were reported to experience a disproportionate burden, as disruption to established dietary routines frequently resulted in behavioural distress and increased reliance on specialist metabolic teams.

In addition to the direct impact on dietary treatment, respondents described a considerable treatment burden associated with maintaining access to medical foods. Families frequently spent substantial time coordinating between GP practices, pharmacies, suppliers, and home-delivery providers to resolve prescribing and supply problems. These activities, often undertaken alongside employment and caregiving responsibilities, resulted in avoidable travel, duplicate or incorrect deliveries, product wastage, and additional financial and logistical pressures.

Difficulties accessing SLPFs also affected participation in everyday life. The unavailability of staple products disrupted school meals and celebrations, limiting opportunities for children to participate alongside their peers. Reduced access to familiar foods increased the complexity of meal planning and diminished families’ confidence in managing dietary treatment away from home.

Collectively, these experiences eroded confidence in the prescribing and supply pathway. Respondents described frustration and stress arising from the perception that reliable access to essential dietary treatments depended on sustained vigilance, repeated intervention and self-advocacy by patients and caregivers. This included repeatedly explaining PKU and its dietary management to non-specialist healthcare professionals, effectively transferring responsibility for navigating and maintaining continuity of prescribed treatment onto patients and families.

Several respondents also suggested that repeated failures undermined motivation to adhere to prescribed dietary treatment, as efforts to maintain metabolic control were compromised by factors beyond their control.

### 3.5. Impact on Metabolic Dietetic Services of Supply Disruptions to Protein Substitute and Specialist Low Protein Foods

Respondents consistently reported that prescribing and supply disruptions imposed a substantial burden on specialist metabolic dietetic services. Managing problems across the prescribing pathway required considerable clinical time, with dietitians frequently correcting prescription errors, liaising with GP practices, pharmacies, manufacturers, and home-delivery providers, and coordinating access to prescribed dietary products.

In addition to resolving routine prescribing problems, metabolic teams were often required to implement contingency measures to maintain continuity of treatment, including identifying alternative supply routes, arranging emergency access to products, and facilitating temporary treatment solutions. These activities extended well beyond the traditional scope of specialist metabolic dietetic practice and diverted clinical expertise away from direct patient care, dietary optimisation, education, service development, and research.

Respondents described repeatedly compensating for failures occurring elsewhere in the prescribing and supply pathway, effectively acting as the final safeguard against interruptions to essential dietary treatment. This reactive workload generated sustained administrative burden, increased professional frustration, and reduced service capacity, with respondents expressing concern that specialist resources were increasingly directed towards mitigating avoidable system failures rather than delivering specialist metabolic care.

**Figure 2:**
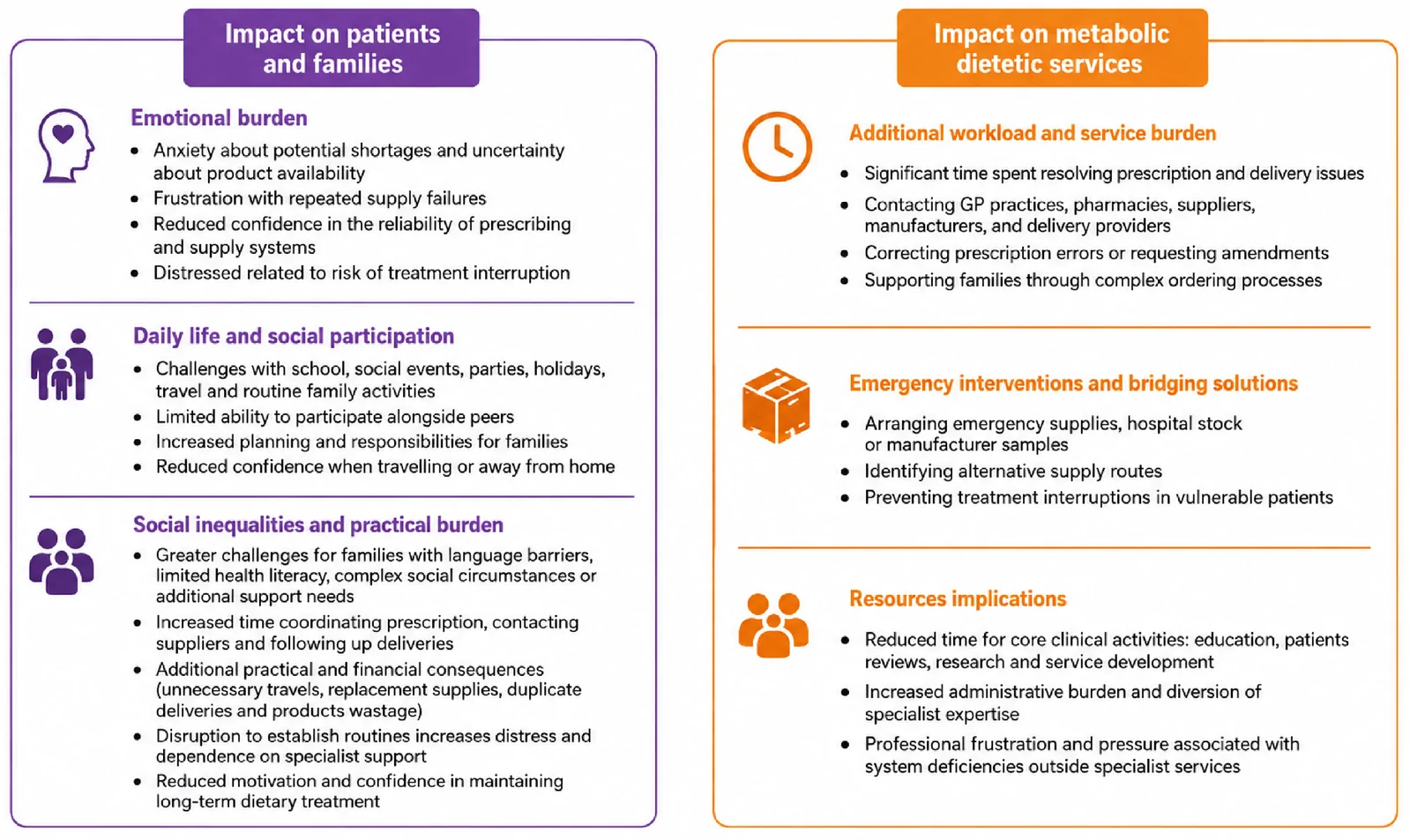
Impact of prescribing issues on patients, families and metabolic dietetic services (figure created by the authors using BioRender and refined using AI-assisted visual editing).

The principal themes describing the impact of prescribing and supply issues on patients, families, and specialist metabolic dietetic services are summarised in Figure 2, while Table 1 presents representative anonymous quotes from dietitians describing these impacts.

## 4. Discussion

This study provides the first comprehensive evaluation of barriers to accessing medical foods within the UK prescribing pathway from the perspective of metabolic dietitians. By capturing the experiences of healthcare professionals directly involved in dietary recommendations, coordinating supply, and maintaining continuity of dietary treatment, this study complements previous research that has focused primarily on patient and caregiver experiences and provides important insight into the system-level origins of access difficulties in routine PKU care. Although patient and caregiver perspectives are essential for understanding the consequences of disrupted access, they may provide less insight into where and why problems arise within the prescribing and supply pathway, how they are managed, and the considerable professional time required to resolve them. Metabolic dietitians are uniquely positioned to identify these challenges because they work across the multiple services and suppliers involved in prescribing and supplying patients with their protein substitutes and SLPFs.

Disruptions in access to specialist dietary products were predominantly associated with failures across the prescribing and supply pathway rather than product shortages, with prescribing processes, inter-organisational communication, dispensing arrangements, home-delivery services, and administrative complexity emerging as the principal barriers. Although SLPFs accounted for the largest proportion of reported incidents, both SLPFs and protein substitutes were associated with substantial disruption to dietary treatment, increased burden for families, and considerable demands on specialist metabolic services.

Notably, two-thirds of reported incidents involved children, emphasising the vulnerability of paediatric patients, who depend on caregivers and healthcare systems to ensure continuity of dietary treatment during critical periods of growth and neurodevelopment. Nevertheless, a substantial proportion of incidents involving adults indicates that prescribing failures extend beyond paediatric care and persist across the life course. This finding is particularly important given that individuals with PKU require lifelong dietary management, including continued access to specialist nutritional products [16,17]. Collectively, these findings highlight the need for robust prescribing and supply systems that support continuity of treatment across all ages and transitions in care.

The predominance of prescribing and administrative failures suggests that interruptions to treatment arise primarily from weaknesses within healthcare delivery rather than limitations in product availability [7,10,11]. Unlike conventional medicines, protein substitutes and SLPFs require coordination between specialist metabolic teams, primary care, community pharmacies, home-delivery providers, and manufacturers [18,19,20]. Each additional interface introduces opportunities for communication failure, prescribing error, or delay [21]. The repeated need for metabolic dietitians to intervene to correct prescriptions and coordinate supply illustrates a pathway that relies heavily on individual effort rather than robust, integrated systems [22].

These findings are consistent with experience from other specialised nutritional prescribing pathways within the NHS. In coeliac disease, access to gluten-free staple foods through primary care has long been affected by national prescribing restrictions and heterogeneous local commissioning policies, resulting in substantial regional variation in eligibility criteria and inequitable access despite dietary exclusion of gluten being the only effective treatment [23,24,25,26,27]. Similarly, home enteral nutrition requires complex coordination between secondary care, primary care, dietitians, pharmacists and homecare providers to ensure appropriate prescribing, supply continuity and monitoring of nutritional treatment [28,29,30,31]. In response, dedicated prescribing pathways, multidisciplinary shared-care models and specialist homecare services have been developed to improve continuity of care and reduce avoidable interruptions to nutritional treatment [11,32,33]. However, important distinctions exist. Unlike gluten-free foods, protein substitutes and SLPFs are not optional dietary supplements or lifestyle products; they constitute the primary treatment for PKU and are indispensable for maintaining metabolic stability [2,20]. Furthermore, compared with enteral home nutrition, individuals with PKU require continual adjustment of multiple dietary products in response to age, growth, metabolic control and personal preferences. This creates a prescribing pathway of considerably greater complexity, making timely access to the correct products particularly critical for maintaining treatment adherence and preventing metabolic deterioration [2]. The experience of other specialised nutritional prescribing pathways suggests that consistent national commissioning arrangements, specialist dietetic oversight and coordinated prescribing processes are essential to minimise unwarranted variation and ensure equitable access to clinically indicated nutritional products [27,31]. Similar principles may be required for PKU to optimise continuity of treatment and reduce prescribing-related inequalities across the UK.

SLPFs appeared particularly vulnerable to disruption. Their role extends beyond nutritional support to facilitating dietary variety, meal planning, normal eating behaviours, and social participation [12,34]. The large number of individual products, combined with changing dietary requirements and patient-specific preferences, inevitably increases the complexity of ordering and supply [35]. Consequently, interruptions in SLPF availability affected not only nutritional management but also family routines, school participation, travel, and quality of life. These findings support recognition of SLPFs as essential therapeutic foods rather than discretionary dietary products [36].

The clinical implications of these failures may be considerable. Respondents reported reduced adherence, deterioration in metabolic control, delays in dietary optimisation, and interruptions to treatment transitions as consequences of prescribing and supply problems. Infants, young children, women planning pregnancy or during pregnancy, and individuals with classical PKU were perceived to be particularly vulnerable, given the importance of uninterrupted dietary management to effective treatment [3,37,38,39].

Although these outcomes were not directly measured in the present study, the reported experiences are consistent with established evidence highlighting the importance of continuous access to protein substitutes and SLPFs for maintaining dietary adherence and metabolic control [3,37,40,41]. These findings highlight that reliable access to essential medical foods should be regarded as a clinical priority and an integral component of safe, effective PKU management.

An equally important finding was the substantial hidden workload generated for specialist metabolic dietetic services. Rather than focusing exclusively on nutritional management, dietitians frequently became responsible for troubleshooting prescriptions, coordinating communication between organisations, arranging emergency supplies, and advocating on behalf of patients. This administrative burden diverted highly specialised clinical expertise away from direct patient care [42,43]. Similar displacement of specialist time has been described in enteral nutrition services, where multidisciplinary teams often compensate for deficiencies within prescribing and supply systems. However, given the limited workforce in inherited metabolic disorders, the opportunity cost is likely to be proportionately greater.

Collectively, these findings highlight the need to redesign current prescribing and supply pathways for specialist dietary products in PKU. Potential approaches include clearer accountability across the prescribing pathway, improved interoperability between specialist and primary care systems, greater flexibility in prescribing, dispensing, and delivery arrangements, and processes that minimise administrative burden for both patients and healthcare professionals. Existing models of integrated home enteral nutrition services, where prescribing, dispensing, delivery, and clinical oversight are coordinated through specialist providers, may provide a useful framework for the provision of specialist dietary products in inherited metabolic disorders. Alternative approaches should also be evaluated, including dietitian-led prescribing, where appropriate governance arrangements exist, to enable specialist clinicians to prescribe directly and reduce avoidable delays in treatment initiation and continuation [44,45]. Similarly, pre-paid card schemes, which have been implemented in some regions to support access to gluten-free foods, may offer a more flexible and patient-centred approach to obtaining specialised low-protein foods while reducing administrative complexity [25]. Such schemes offset the additional costs associated with therapeutic diets and may provide greater choice for patients and families [46,47]. Although these approaches have not been evaluated within PKU prescribing pathways and would require formal evaluation before implementation, they may offer useful models for reducing administrative complexity, improving responsiveness, and promoting more equitable access to specialist dietary products. Reducing unwarranted regional variation should also remain a priority to ensure consistent access to essential dietary treatments irrespective of geographical location. Potential service-level approaches to improving prescribing and supply pathways for protein substitutes and SLPFs are summarised in Table 2.

This study has several strengths. To our knowledge, it provides the first national evaluation of barriers to accessing protein substitutes and SLPFs from the perspective of specialist metabolic dietitians responsible for coordinating lifelong dietary management in PKU. Importantly, our findings corroborate and extend previous reports of persistent difficulties obtaining prescribed dietary products and navigating non-specialist healthcare services, demonstrating that these barriers remain embedded within routine care despite having been recognised for many years [7,12,18,33]. By capturing the experiences of specialist clinicians working across the patient pathway, this study highlights that difficulties with access are not isolated events but reflect recurring challenges within prescribing, procurement and supply systems that may undermine continuity of dietary treatment. Respondents had direct clinical experience of the prescribing pathway across primary care, pharmacy and home-delivery services, providing insights into system-level failures that are unlikely to be captured through routine administrative datasets. The inclusion of free-text responses enabled detailed exploration of the clinical, psychosocial and service-level consequences of prescribing failures, while evaluation of both protein substitutes and SLPFs allowed comparison of shared and product-specific barriers across the prescribing pathway.

Several limitations should be acknowledged. The findings are based on self-reported experiences of metabolic dietitians and therefore reflect clinically recognised incidents rather than the true frequency of prescribing or supply failures across the healthcare system. Consequently, more complex or clinically significant events may be overrepresented. As a cross-sectional survey, the study cannot establish causal relationships between prescribing failures and clinical outcomes, nor quantify their direct effect on metabolic control or health-related quality of life. In addition, the study was conducted within the UK National Health Service, where prescribing and reimbursement arrangements for specialist dietary products differ from those in other healthcare systems, potentially limiting international generalisability. Nevertheless, the systemic challenges identified, including fragmented prescribing pathways, poor communication between providers, and administrative complexity, are likely to be relevant to the provision of specialised nutritional therapies beyond PKU.

## 5. Conclusions

In conclusion, this study demonstrates that barriers within the UK prescribing and supply pathway for protein substitutes and SLPFs are common, multifactorial, and have important consequences for patients, families, and metabolic services. Problems were predominantly associated with prescribing processes, communication failures, and the complexity of the supply pathway rather than confirmed product shortages. These disruptions threatened continuity of dietary treatment, increased burden on patients and families, created avoidable clinical risks, and generated substantial additional workload for metabolic dietitians. The findings highlight the need to evaluate alternative prescribing and supply models and provide a basis for service-level interventions aimed at reducing avoidable delays, administrative burden, and unwarranted variation in access. A more integrated and flexible pathway is required, with clearer responsibilities across specialist services, primary care, pharmacies, suppliers, and home-delivery providers; improved communication between these interfaces; standardised procedures; and additional support for patients who are particularly vulnerable to disruption.

Protein substitutes and SLPFs are essential components of dietary treatment for PKU, rather than optional dietary products, and prescribing and supply systems should reflect their clinical importance. Ensuring timely and reliable access throughout life is fundamental to maintaining continuity of dietary treatment and should be regarded as a core component of high-quality PKU care.

## Figures and Tables

**Table 1:** Representative anonymous quotes from dietitians describing the impact of prescribing issues on patients, families, and metabolic dietetic services.

Theme	Examples of Quotes
Impact of prescribing issues on patients and families	Patient: *“I’ve stopped having breakfast as I can’t find anything that’s low enough to eat. I feel tired and hungry all the time.”* Patient: *“I’m having to ring the GP each time I want to access low protein foods. I then go to the chemist and most of the time they say they haven’t received the prescription authorised by the GP. I’m then told to go back to the GP to get the prescription. I don’t feel it is reasonable having to constantly contact the pharmacy to chase the prescription. The issues are not getting resolved and I feel like I’m having to act as a go-between, between the GP and chemist.”* Dietitian: *“Patient lacks the confidence to contact the GP about prescription issues and often feels they are being a nuisance and not being taken seriously.”* Dietitian: *“Patient is stressed due to the lack of supplies of protein substitute and low protein foods. It is very hard for her to follow the low protein diet without supplies. She has made an appointment to speak to her GP to try and sort out the issue. In the meantime, she has been buying gluten free products that are lower in protein but still higher than what she requires, so going over her protein exchanges daily. The is likely to worsen her Phe control.”* Dietitian: *“Anxiety, additional workload and unnecessary stress for patient having to go back and forwards to the pharmacy to chase her prescriptions. The patient has worsened Phe control due to shortfall in supply of protein substitute.”* Dietitian: *“Patient has autism and is unable to obtain the correct protein substitute flavour. This causes anxiety when they are running low of this supplement due to delays.”* Dietitian: *“Child ran out of basic low protein foods. Child’s appetite increased. Also wanting more low protein milk in cold weather. Mother lacked flexibility to order more according to child’s appetite or season of the year. Child was hungry and his mother had to ration the child’s foods.”*
Impact of prescribing issues on metabolic dietetic services	Dietitians: *“We are being used as the messengers by home delivery companies—but this is taking substantial time. However, we cannot leave a child without any protein substitute.”* Dietitians: *“Dietitians had to arrange emergency supplies and increase orders with home delivery companies because of repeated delays in the delivery of low protein foods.”* Dietitians: *“A lot of extra dietetic time was required. The chemist was unable to access low protein foods. The patient requested her low protein items from the pharmacy at least three weeks ago but she has still not received any update. We have been trying to contact the pharmacy without success. We had to organise emergency supplies.”*

**Table 2:** Potential approaches to improve prescribing and supply pathways for protein substitutes and SLPFs in PKU.

Main Barrier	Potential Approach
GP prescribing and prescription processing	Develop clearer prescribing guidance and standardised processes, including prescription templates specifying product name, formulation, quantity, and frequency. Clarify prescribing responsibilities and strengthen communication between primary and specialist care, including communication of specialist prescribing recommendations. Establish clear procedures for amending prescriptions when requirements change with age, growth, or dietary needs.
Pharmacy-related barriers	Improve pharmacy awareness of PKU-specific products and provide clear guidance on product identification, ordering, and dispensing. Establish direct communication channels between pharmacies, metabolic teams, and suppliers, with defined escalation pathways for unresolved prescription or dispensing problems. Strengthen coordination when products are unavailable locally. Develop more reliable and coordinated delivery systems, with timely communication regarding delays, substitutions, or changes to scheduled deliveries. Establish clear responsibility for stock management and procedures for urgent or missed deliveries. Introduce proactive monitoring of recurrent delivery problems and greater flexibility to accommodate changes in product requirements and quantities.
Home delivery and supply management	Develop more reliable and coordinated delivery systems, with timely communication regarding delays, substitutions, or changes to scheduled deliveries. Establish clear responsibility for stock management and procedures for urgent or missed deliveries. Introduce proactive monitoring of recurrent delivery problems and greater flexibility to accommodate changes in product requirements and quantities.
Ordering systems and administrative complexity	Streamline processes involving multiple organisations and improve interoperability and information sharing across prescribing, dispensing, and delivery systems. Reduce unnecessary administrative steps and duplication, while increasing flexibility in prescribing, dispensing, and delivery arrangements. Establish clear procedures for provider or system transitions and mechanisms to identify and address recurrent supply problems.
Fragmented responsibility and additional dietetic workload	Define responsibilities across the prescribing and supply pathway and establish clear escalation routes so that recurrent problems are managed by the appropriate organisation rather than individual dietitians. Develop mechanisms to monitor prescribing- and supply-related incidents and reduce avoidable administrative workload for metabolic teams. Where appropriate, consider specialist or dietitian-led prescribing within appropriate governance and competency frameworks.

## Data Availability

The data supporting the findings of this study are available from the corresponding author upon reasonable request. The data are not publicly available due to ethical reasons.

## Appendix A

**Table A1:** Questionnaire.

Questions
Tell us about the individual with PKU who experienced the prescription issue? Child Adult Other (specify)
2. What dietary products did the patient have difficulty with? Protein substitute Low protein foods Both protein substitute and low protein foods
3. Please describe (in as much detail as you can) what the problem was? Was this an issue with the GP, pharmacy or home delivery company? Was there an issue with the quality of the products received (e.g., damaged products)?
4. Please tell us what effects this has had on the individual with PKU or their caregivers? (e.g., Frustration, worsened blood Phe control)
5. Anything else you want to tell us?
